# Region-Based Analyses of Existing Genome-Wide Association Studies Identifies Novel Potential Genetic Susceptibility Regions for Glioma

**DOI:** 10.1158/2767-9764.CRC-24-0385

**Published:** 2024-11-12

**Authors:** Karen Alpen, Robert J. Maclnnis, Claire M. Vajdic, John Lai, James G. Dowty, Eng-Siew Koh, Elizabeth Hovey, Rosemary Harrup, Tuong L. Nguyen, Shuai Li, David Joseph, Geza Benke, Pierre-Antoine Dugué, Melissa C. Southey, Graham G. Giles, Anna K. Nowak, Katharine J. Drummond, Daniel F. Schmidt, John L. Hopper, Miroslaw K. Kapuscinski, Enes Makalic

**Affiliations:** 1 Centre for Epidemiology and Biostatistics, Melbourne School of Population and Global Health, The University of Melbourne, Parkville, Australia.; 2 Cancer Epidemiology Division, Cancer Council Victoria, Melbourne, Australia.; 3 The Kirby Institute, UNSW Sydney, Sydney, Australia.; 4 Australian Genome Research Facility, St Lucia, Australia.; 5 South Western Sydney Clinical School, Faculty of Medicine, University of New South Wales, Liverpool, Australia.; 6 Liverpool and Macarthur Cancer Therapy Centres, Liverpool Hospital, Liverpool, Australia.; 7 Ingham Institute for Applied Medical Research, Liverpool, Australia.; 8 Nelune Comprehensive Cancer Centre, Prince of Wales Hospital, Sydney, Australia.; 9 Faculty of Medicine, Prince of Wales Clinical School UNSW Sydney, Sydney, Australia.; 10 Royal Hobart Hospital, Hobart, Australia.; 11 University of Tasmania, Hobart, Australia.; 12 Precision Medicine, School of Clinical Sciences at Monash Health, Monash University, Clayton, Australia.; 13 Department of Public Health and Primary Care, Centre for Cancer Genetic Epidemiology, University of Cambridge, Cambridge, United Kingdom.; 14 Murdoch Children’s Research Institute, Royal Children’s Hospital, Parkville, Australia.; 15 Department of Medicine and Surgery, The University of Western Australia, Perth, Australia.; 16 School of Public Health and Preventative Medicine, Monash University, Clayton, Australia.; 17 Department of Clinical Pathology, Melbourne Medical School, The University of Melbourne, Parkville, Australia.; 18 Medical School, University of Western Australia, Crawley, Australia.; 19 Department of Neurosurgery, Royal Melbourne Hospital, Parkville, Australia.; 20 Department of Surgery, University of Melbourne, Parkville, Australia.; 21 Faculty of Information Technology, Monash University, Clayton, Australia.

## Abstract

Glioma is a rare and debilitating brain cancer with one of the lowest cancer survival rates. Genome-wide association studies have identified 34 genetic susceptibility regions. We sought to discover novel susceptibility regions using approaches that test groups of contiguous genetic markers simultaneously. We analyzed data from three independent glioma studies of European ancestry, GliomaScan (1,316 cases/1,293 controls), Australian Genomics and Clinical Outcomes of Glioma Consortium (560 cases/2,237 controls), and Glioma International Case-Control Study (4,000 cases/2,411 controls), using the machine learning algorithm DEPendency of association on the number of Top Hits and a region-based regression method based on the generalized Berk–Jones (GBJ) statistic, to assess the association of glioma with genomic regions by glioma type and sex. Summary statistics from the UCSF/Mayo Clinic study were used for independent validation. We conducted a meta-analysis using GliomaScan, Australian Genomics and Clinical Outcomes of Glioma Consortium, Glioma International Case-Control Study, and UCSF/Mayo. We identified 11 novel candidate genomic regions for glioma risk common to multiple studies. Two of the 11 regions, 16p13.3 containing *RBFOX1* and 1p36.21 containing *PRDM2*, were significantly associated with female and male glioma risk respectively, based on the results of the meta-analysis. Both regions have been previously linked to glioma tumor progression. Three of the 11 regions contain neurotransmitter receptor genes (7q31.33 *GRM8*, 5q35.2 *DRD1*, and 15q13.3 *CHRNA7*). Our region-based approach identified 11 genomic regions that suggest an association with glioma risk of which two regions, 16p13.3 and 1p36.21, warrant further investigation as genetic susceptibility regions for female and male risk, respectively. Our analyses suggest that genetic susceptibility to glioma may differ by sex and highlight the possibility that synapse-related genes play a role in glioma susceptibility.

**Significance:**

Further investigation of the potential susceptibility regions identified in our study may lead to a better understanding of glioma genetic risk and the underlying biological etiology of glioma. Our study suggests sex may play a role in genetic susceptibility and highlights the importance of sex-specific analysis in future glioma research.

## Introduction

Gliomas are the most common primary malignant tumors of the adult brain. Glioma types differ in both histology and molecular genetic features, with the major type, glioblastoma isocitrate dehydrogenase (IDH) wild type (GBM), having one of the shortest median cancer survival times of less than 15 months (1). Although the survival time is longer for lower-grade astrocytoma and oligodendroglioma, these tumors are still ultimately fatal. The average annual age-adjusted incidence of glioma is approximately 6.0 per 100,000 people and is 1.4 times higher in men than in women (1). This sex difference in incidence exists across adult age groups and ethnicities (1, 2). High levels of ionizing radiation are the only consistently validated environmental risk factor for glioma (3), and there is a protective association with some allergies (4, 5).

Family aggregation studies report an approximately two-fold increase in glioma risk for individuals with an affected first-degree relative (6, 7). Several rare inherited cancer syndromes (including retinoblastoma, neurofibromatosis 1, Li-Fraumeni syndrome, tuberous sclerosis, Lynch/Turcot syndrome, and melanoma-neural system tumor syndrome) are associated with an increased risk of glioma, but they account for only 1% to 2% of adult glioma cases (5). Thirty-four genomic susceptibility regions have been identified by previous genome-wide association studies (GWAS) with some associations specific to certain glioma types or grades (8–19). Associations with variants in known glioma susceptibility regions account for less than 40% of the familial risk (8), leaving a considerable proportion of the familial risk unexplained.

Identifying the missing heritability of glioma has been hindered by the small sample size and limitations of conventional GWAS analyses. Conventional GWAS analysis treats each genetic variant (single nucleotide polymorphism, or SNP) as an independent test and requires stringent genome-wide significance thresholds (typically 5 × 10^−8^) to account for multiple testing of millions of SNPs. SNPs associated with the disease are usually markers of a causal variant, and not the causal variant itself (20). Increasing the sample size can increase the power to detect these markers; however, glioma sample sizes are small because of its low population incidence, short survival, and debilitating nature. The largest glioma GWAS was a meta-analysis of 12,496 cases and 18,190 controls (8). Consequently, conventional GWAS analysis may be underpowered to detect new susceptibility variants of glioma. Thus, discovering the missing heritability of glioma requires the application of novel statistical methods to existing datasets.

The primary aim of this study was to conduct a region-based GWAS to identify novel susceptibility regions for glioma risk. This study used two region-based GWAS methods, a supervised machine learning algorithm called DEPTH (DEPendency of association on the number of Top Hits; ref. 21) and logistic regression with the generalized Berk–Jones statistic (GBJ; ref. 22). We used DEPTH as a hypothesis-generating tool to prioritize candidate genetic regions that were tested for their association with glioma risk using GBJ. Both methods consider a set of SNPs within a region, rather than analyzing each SNP individually, allowing the aggregation of weak association signals from nearby SNPs. DEPTH uses decision tree analysis, which is a machine learning method that learns simple decision rules from the data. It uses a series of overlapping sliding windows to traverse the genome and analyzes groups of contiguous SNPs within each window, considering the correlation and interaction between SNPs (Supplementary Fig. S1). Decision tree analysis makes no assumption about the distribution of the data and can model nonlinear relationships. DEPTH analyses have identified new putative risk-associated regions for prostate (23) and colorectal cancer (24) that have not been detected using conventional GWAS methods. The GBJ is a modified version of the standard Berk–Jones statistic (25), which accounts for the correlation between individual SNPs in a predefined region or gene. GBJ offers good power to detect moderately sparse effects while still performing well when SNP signals are extremely sparse (22).

The secondary aim of our study was to identify sex differences in genetic susceptibility. The brain differs by sex in structure, function, and gene expression (26, 27) and there are sex differences in glioma incidence, survival, tumor molecular characteristics, and therapy responses (28). Two sex-specific risk variants have been identified in regions 3p21.31 and 7p11.2, whereas variants in the 8q24.21 region are associated with non-GBM risk for both sexes but with a greater risk for the female sex (11, 29).

We conducted a GWAS by glioma type and sex using three independent glioma studies: GliomaScan consortium (hereafter referred to as GliomaScan; ref. 30; 1,316 cases and 1,293 controls), the Australian Genomics and Clinical Outcomes of Glioma Consortium (AGOG; ref. 29; 560 cases and 2,237 controls), and Glioma International Case-Control Study (GICC; ref. 31; 4,000 cases and 2,411 controls). We used DEPTH as a genome-wide screen to identify candidate susceptibility regions, for which we performed a risk association analysis using GBJ. Summary statistics from the University of California San Francisco and Mayo Clinic study (UCSF/Mayo; ref. 9) were used for independent validation. We then performed a meta-analysis (GliomaScan, AGOG, GICC, and UCSF/Mayo) for candidate regions of interest.

## Materials and Methods

### Ethics statement

Ethical approval for research into genetic susceptibility to glioma was obtained from the University of Melbourne School of Population and Global Health (MSPGH) Human Ethics Advisory Group (HEAG) on March 26, 2020 (1954154.2). The collection of AGOG patient samples and clinical information was obtained with written informed consent. Ethical review board approval was obtained from the Cancer Council Victoria Human Research Ethics Committee (HREC) on June 14, 2012 (HREC1208), and HRECs were appropriate for the multiple clinical recruitment sites. Ethical approval for the GliomaScan and GICCs was previously described (30, 31).

### Data

GliomaScan and GICC data were obtained from the Database of Genotypes and Phenotypes (dbGaP; phs000652.v1.p1 and phs001319.v1.p1, respectively; refs. 30, 31), and AGOG is an Australian hospital-based glioma study. Details of the data collection and genotyping methods for all three studies have been previously described (29–31). The AGOG case data were collected between January 2013 and November 2017; GliomaScan data, between 1997 and 2011; and GICC data, between 2010 and 2013. The sample sizes for the association analyses were as follows: 1,316 cases and 1,293 controls for GliomaScan; 560 cases and 2,237 controls for AGOG; and 4,000 cases and 2,411 controls for GICC. The AGOG controls included unaffected women participating in the Australian Mammography Density Twin and Sister Study and unaffected men participating in the Lifestyle and Genetic Risk Factors for Prostate Cancer Study as previously described (29). The Illumina Infinium OncoArray-500K BeadChip array was used for genotyping GICC and AGOG, whereas GliomaScan was genotyped using the Illumina Human660W array (29–31). As previously described (31), the array used for GICC was customized to include SNPs in genes previously implicated in glioma risk.

The characteristics of the sample data by glioma type (GBM, non-GBM, astrocytoma, or oligodendroglioma) and sex are summarized in Table 1. Glioma-type information was unavailable for GICC data. The same controls were used for the all-glioma and glioma-type analyses. The previously described UCSF/Mayo summary statistics (2,141 cases and 1,889 controls; ref. 9) were used for independent validation (Table 1; Supplementary Table S1).

**Table 1:** Age and sex characteristics of the discovery datasets (GliomaScan, AGOG, and GICC) and validation dataset (UCSF/Mayo) by glioma type

	Discovery											Validation		
	GliomaScan					AGOG					GICC	UCSF/Mayo summary statistics		
	All glioma	GBM^a^	Non-GBM^b^	Astro^c^	Oligo^d^	All glioma	GBM^a^	Non-GBM^b^	Astro^c^	Oligo^d^	All glioma	All glioma	IDHwt	IDHmut
Combined sex														
No. of cases (% of all glioma cases)	1,316 (100%)	703 (53%)	493 (37%)	181 (14%)	136 (10%)	560 (100%)	370 (66%)	179 (32%)	83 (15%)	46 (8%)	4,000	2,141 (100%)	698 (33%)	622 (29%)
Cases mean age–years (SD)	55.3 (15.6)	60.9 (12.4)	45.6 (15.0)	48.4 (16.1)	44.4 (13.5)	54.7 (14.5)	59.8 (10.9)	44.0 (15.3)	47.9 (15.9)	42.6 (12.9)	51.7 (14.3)	Refer to Supplementary Table S1		
No. of controls	1,293	1,293	1,293	1,293	1,293	2,237	2,237	2,237	2,237	2,237	2,411	1,889	1,889	1,889
Controls mean age–years (SD)	55.9 (15.0)	55.9 (15.0)	55.9 (15.0)	55.9 (15.0)	55.9 (15.0)	58.6 (8.6)	58.6 (8.6)	58.6 (8.6)	58.6 (8.6)	58.6 (8.6)	54.6 (14.0)	Refer to Supplementary Table S1		
Male														
No. of cases (% of all glioma cases)	749 (100%)	416 (56%)	274 (37%)	108 (14%)	65 (9%)	346 (100%)	242 (70%)	97 (28%)	50 (14%)	23 (7%)	2,401	1,251 (100%)	430 (34%)	361 (29%)
Cases mean age–years (SD)	56.0 (15.2)	60.8 (12.3)	46.5 (15.1)	48.4 (15.6)	45.5 (13.9)	56.1 (14.0)	60.1 (11.3)	46.0 (15.2)	51.3 (15.3)	43.4 (12.9)	52.2 (14.2)	Refer to Supplementary Table S1		
No. of controls	687	687	687	687	687	962	962	962	962	962	1,377	1,134	1,134	1,134
Controls mean age–years (SD)	56.4 (14.5)	56.4 (14.5)	56.4 (14.5)	56.4 (14.5)	56.4 (14.5)	62.4 (7.1)	62.4 (7.1)	62.4 (7.1)	62.4 (7.1)	62.4 (7.1)	55.7 (13.6)	Refer to Supplementary Table S1		
Female														
No. of cases (% of all glioma cases)	567 (100%)	287 (51%)	219 (39%)	73 (13%)	71 (13%)	214 (100%)	128 (60%)	82 (38%)	33 (15%)	23 (11%)	1,599	890 (100%)	268 (30%)	261 (29%)
Cases mean age–years (SD)	54.5 (16.0)	61.0 (12.7)	44.4 (14.9)	48.3 (16.9)	43.4 (13.1)	52.4 (15.1)	59.4 (10.2)	41.6 (15.2)	42.8 (15.7)	41.7 (13.1)	50.9 (14.4)	Refer to Supplementary Table S1		
No. of controls	606	606	606	606	606	1,275	1,275	1,275	1,275	1,275	1,034	755	755	755
Controls mean age–years (SD)	55.2 (15.7)	55.2 (15.7)	55.2 (15.7)	55.2 (15.7)	55.2 (15.7)	55.7 (8.5)	55.7 (8.5)	55.7 (8.5)	55.7 (8.5)	55.7 (8.5)	53.2 (14.3)	Refer to Supplementary Table S1		
Ratio of male to female cases	1.3	1.4	1.3	1.5	0.9	1.6	1.9	1.2	1.5	1.0	1.5	1.4	1.6	1.4

Abbreviation: SD, standard deviation.

a Glioblastoma (GBM) includes disease classification codes 9440, 9441, and 9442.

b Non-GBM includes glioma classification codes not designated as GBM with the exception of the generic glioma classification code 9380.

c Astrocytoma includes disease classification codes 9400 and 9401.

d Oligodendroglioma includes disease classification codes 9450 and 9451.

#### Quality control

Samples were excluded for the following reasons: genotyping call rate <95%, duplicates, sex discordance, missing phenotype data, non-European ethnicity, related individuals, divergent European ancestry, duplicate samples among studies, and study ineligibility (Supplementary Tables S2, S3A, S3B, and S4). AGOG case samples removed for study ineligibility did not meet the age at diagnosis/enrolment criteria or were deemed ineligible based on the final morphological diagnosis. Relatedness was measured by pair-wise identity by descent (IBD) using PLINK (32) and any individuals with an IBD >0.1875 (halfway between a second- and third-degree relative) were excluded from further analysis. Exclusion because of divergent European ancestry was made to mitigate bias arising from the population structure. Divergent European ancestry was defined with reference to the 1000 Genomes Project European reference population (EUR; ref. 33). The study samples were merged with the 1000 Genome Project reference, and genetic ancestry was visualized on a graph of the first two principal components (PC). The study samples that appeared visually as outliers from the EUR reference cluster were removed (Supplementary Fig. S2).

Quality control was performed separately for each study. SNPs were excluded if they had a genotyping call rate <95%, had significant variation in call rate between cases and controls (*P* < 1 × 10^−5^), had a minor allele frequency of <0.01, or if their distribution for controls deviated from the expected Hardy–Weinberg proportions (*P* < 1 × 10^−5^). To mitigate sex-specific genotyping errors, any SNPs whose distribution deviated from the expected Hardy–Weinberg proportions within male-only or female-only controls were removed. All multi-allelic SNPs, insertions, deletions, and copy number variations were removed. The UCSC LiftOver tool (34) converted the genome coordinates of GliomaScan from UCSC hg18 (NCBI build 36) to UCSC hg19 (NCBI build 37) to match the genome assemblies of AGOG and GICC. The remaining genotyped SNPs were used to infer the alleles of other common variants by imputation using the Michigan Imputation Server (Minimac4; ref. 35). The reference panel used for phasing by Eagle v2.4 was the Haplotype Reference Consortium (HRC) version r1.1 2016 (GRCh37/hg19), which consisted of 64,940 haplotypes of predominantly European ancestry (36). Imputed SNPs that were multi-allelic, had an Rsq <0.30 or a minor allele frequency of <0.01 were removed. After quality control, the final number of SNPs available for analysis, including the sex chromosomes, was 7,829,427 (523,032 genotyped and 7,306,395 imputed) for GliomaScan, 7,792,444 (348,745 genotyped and 7,443,699 imputed) for AGOG, and 7,827,226 (417,849 genotyped and 7,409,377 imputed) for GICC. For the analysis of aggregated data, GliomaScan, AGOG, and GICCs were aggregated using PLINK (32). SNPs not available in all three studies or with >5% missing data in the aggregated dataset were removed, leaving 7,395,906 autosomal SNPs for the aggregated analysis.

#### Sample stratification for subanalyses

GliomaScan and AGOG tumors were stratified by GBM, non-GBM, astrocytoma, and oligodendroglioma, according to the WHO classification of central nervous system tumor current at the time of data collection. Stratification based on IDH gene mutation and 1p/19q codeletion status was not possible, as these molecular markers were not consistently reported during the period of recruitment. Histopathology was used as a proxy for IDH mutation status, with GBM being primarily IDH wild type (IDHwt), astrocytoma mostly IDH mutant, 1p/19q intact, and oligodendroglioma IDH mutant, 1p/19q codeleted (37). GBM included the International Classification of Diseases morphological codes 9440/3 glioblastoma NOS, 9441/3 giant cell glioblastoma, and 9442/3 gliosarcoma. Non-GBM included all tumors other than those classified as GBM, with the exclusion of 9380/3 malignant glioma. Oligodendroglioma included the International Classification of Diseases codes of 9450/3 oligodendroglioma NOS and 9451/3 oligodendroglioma anaplastic. Astrocytomas included 9400/3 astrocytoma NOS and 9401/3 astrocytoma anaplastic. Glioma and all types were further stratified by sex. Information on glioma type for GICC is unavailable. Previously described UCSF/Mayo summary statistics data (9) were stratified by molecular type (IDHwt and IDH mutant).

### Statistical analysis

**Figure 1:**
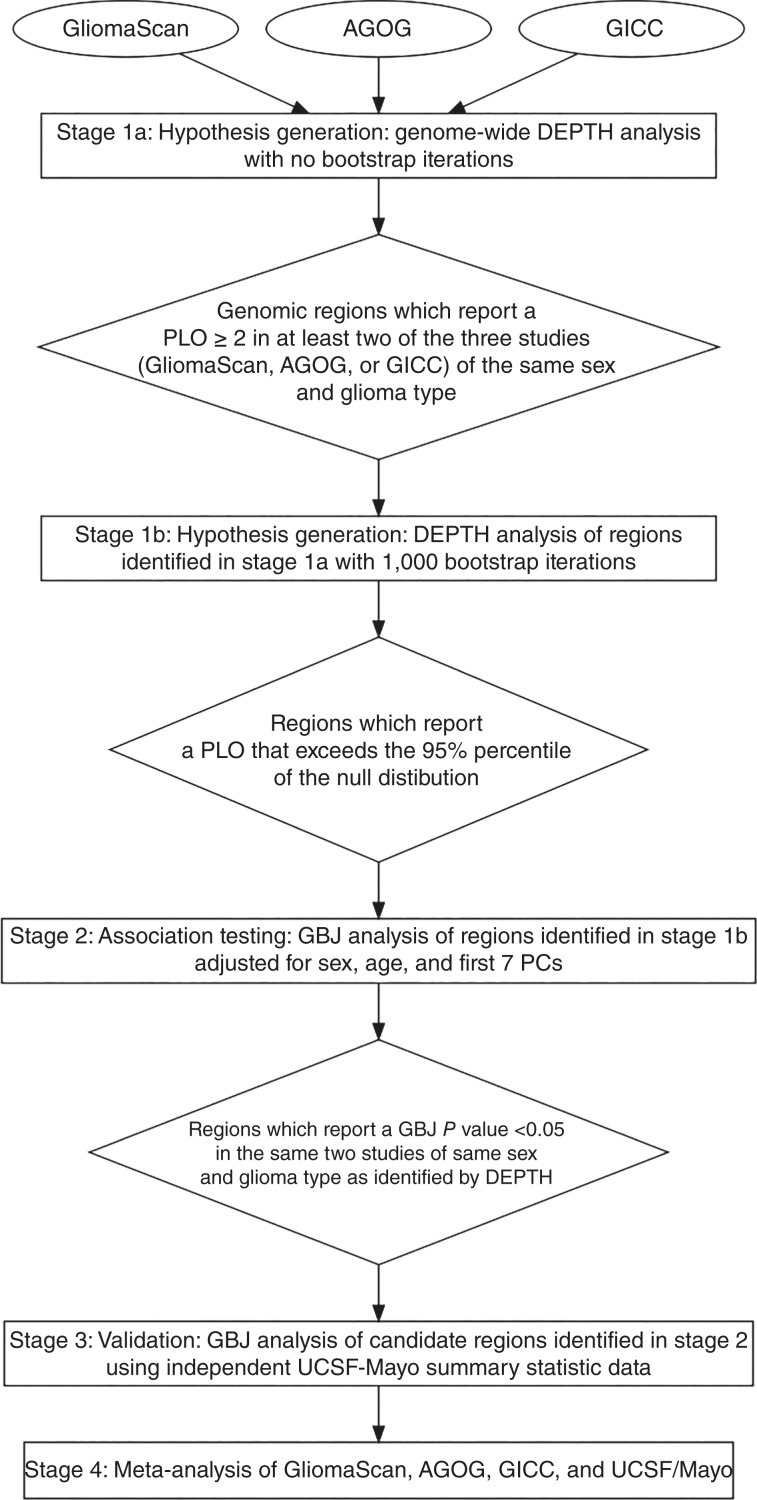
Methodology flowchart. An outline of the stages used to identify potential glioma risk regions. PLO, Bayesian posterior log odds in favor of association; PC, principal component.

Our methodology for the discovery of novel susceptibility regions comprises four stages: (i) hypothesis generation, (ii) association testing, (iii) validation, and (iv) meta-analysis (see Fig. 1).

#### Stage 1: Hypothesis generation using GliomaScan, AGOG, and GICC

We used the in-house developed tool, DEPTH, to jointly analyze groups of contiguous SNPs. DEPTH does not adjust for covariates and was used as a hypothesis-generating tool to prioritize candidate regions for further risk association testing in Stage 2. DEPTH traverses the genome in a series of overlapping sliding windows. This genome-wide screen excluded the X chromosome for combined sex data, as DEPTH does not compensate for the dosage difference between the sexes. DEPTH fitted a Bayesian decision tree and generated a measure of association, called the Bayesian posterior log-odds in favor of association (PLO), for each window of SNPs. The null hypothesis was that no SNP in the window was associated with glioma risk. The distribution of the test statistics under the null hypothesis was approximated using bootstrapping. The DEPTH algorithm was applied to the three studies (GliomaScan, AGOG, and GICC) separately, with a window length of 100,000 base pairs and no bootstrapping. Regions with a DEPTH PLO of ≥2.0 (100 times more likely to be associated with glioma) in at least two of the three studies of the same glioma type and sex, were selected for bootstrapping with 1,000 iterations. A threshold of PLO of ≥2.0 was chosen to mitigate false positives. A previous study (23) found that the 95th percentile of the null distribution approximates a PLO of 1.0 (10 times more likely to be associated with glioma) for each DEPTH window across the genome; therefore, a PLO of ≥2.0 is likely to be above the 95th percentile of the null distribution. Regions with a PLO of >95th percentile of the null distribution after 1,000 bootstrap iterations were selected for risk association testing using GBJ software.

#### Stage 2: Region-based association testing using GliomaScan, AGOG, and GICC

GBJ is a logistic regression set-based inference method that tests the association between a group of SNPs and the outcome (22). GBJ was applied to the selected susceptibility regions from stage 1 to test for their association with glioma or glioma type, adjusting for sex (where applicable), age, and the first seven PCs. The first seven PCs were identified by principal component analysis, conducted using PLINK (32) with default settings. GBJ analysis was conducted on data pruned to remove markers in linkage disequilibrium (LD). Pruning was performed using PLINK (32) under default settings. The regions that reported a GBJ *P* value of <0.05 in the same two studies of the same glioma type and sex as identified by DEPTH were deemed candidate glioma susceptibility regions for further investigation in stage 3.

#### Stage 3: Validation using UCSF/Mayo

GBJ was applied to the candidate susceptibility regions identified in stage 2 using the summary statistics provided by the UCSF/Mayo. When using summary statistics, GBJ requires estimates of LD for each region being tested. We used the 1000 Genomes Project European reference population (33) for LD estimation.

#### Stage 4: Meta-analysis (GliomaScan, AGOG, GICC, and UCSF/Mayo)

A logistic regression analysis using PLINK v2.00a2LM AVX2 (32) adjusted for seven PCs, age, and sex (where applicable) was applied to the candidate susceptibility regions identified in stage 2 across GliomaScan, AGOG, and GICC. A meta-analysis of AGOG, GliomaScan, GICC, and UCSF/Mayo summary statistics was conducted using the weighted sum of the *Z*-score fixed-effects model (38) in PLINK. For those candidate susceptibility regions identified by a sex-specific dataset, a logistic regression analysis and a meta-analysis were conducted for the same genomic region but for the opposite sex to evaluate the sex difference in risk effect size of any SNPs that were significantly associated with glioma risk. The sex difference parameter was estimated by $\beta_{\text{Difference}}\;=\;\beta_{\text{Female}}\;-\;\beta_{\text{Male}\;}$ and the standard error of the sex difference was calculated using ${\text{SE}}_{\text{Difference}}=\;\sqrt{{\text{SE}}_{\text{Male}}^{2}+{\text{SE}}_{\text{Female}}^{2}}$. We also applied the set-based tests GBJ and MAGMA (39) to the meta-analysis results for the 11 candidate susceptibility regions using 1000 genome as a reference for LD estimation. The results of the MAGMA analysis were compared with those of GBJ to ensure consistency of results across different region-based analysis methods.

#### GBJ analysis of aggregated data (GliomaScan, AGOG, and GICC)

For the regions identified in stage 2, we applied the GBJ test using the GliomaScan-AGOG-GICC or GliomaScan-AGOG aggregated data to determine whether the region’s risk association was maintained in the aggregated study relative to the individual studies. The analysis used pruned data adjusted for sex, age, seven PCs, and study.

#### DEPTH analysis of known susceptibility regions

We applied DEPTH to the 34 known glioma susceptibility regions and measured the PLO score for each known susceptibility region across all studies to determine DEPTH’s ability to detect known susceptibility regions.

#### Identification of genes within candidate susceptibility regions

**Figure 2:**
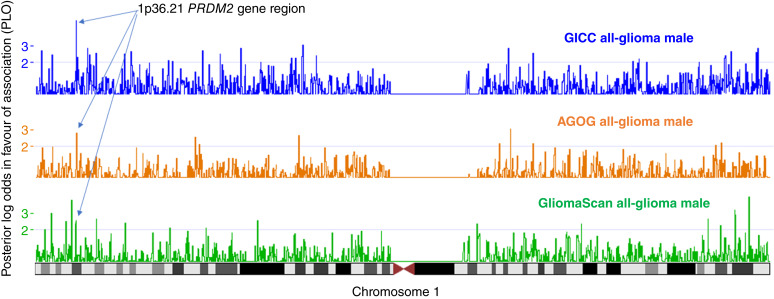
DEPTH results for chromosome 1. Visualization of the DEPTH posterior log-odds in favor of association (PLO) score on the UCSC genome browser (https://genome.ucsc.edu/) for chromosome 1 for GICC (blue), AGOG (orange), and GliomaScan (green). The arrows mark the 1p36.21 *PRDM2* gene region, which reported PLO > 2.0 for all-glioma male data for all three studies.

**Figure 3:**
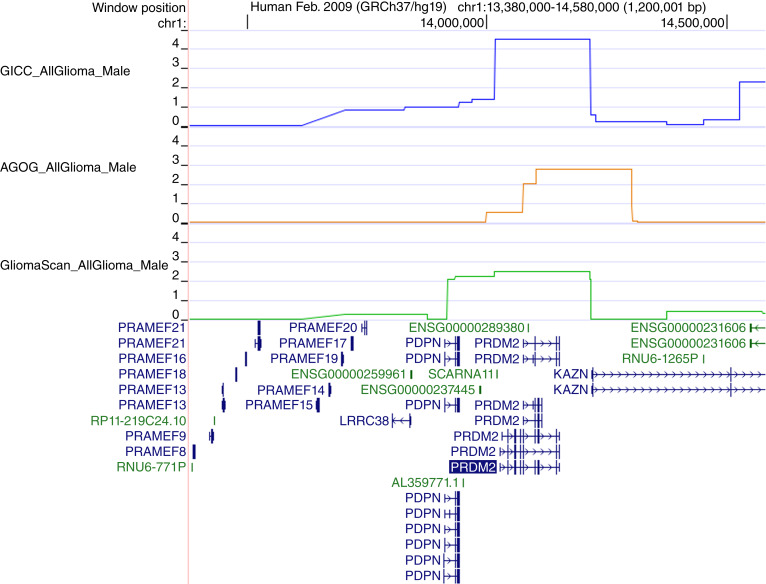
DEPTH results for the 1p36.21 region. DEPTH PLO score for GICC (blue), AGOG (orange), and GliomaScan (green) for the 1p36.21 *PRDM2* gene region for all-glioma male from the UCSC genome browser (https://genome.ucsc.edu/). The PLO score is on the *y*-axis.

The DEPTH PLO scores were uploaded to the UCSC genome browser (http://genome.ucsc.edu/; ref. 34) for visualization (see examples in Figs. 2 and 3), and the genes located within the candidate susceptibility regions from stage 2 were obtained. Known associations of the genes with regulation of glioma tumor cell proliferation; other cancers; and neurodevelopment, neurological disorders, or synapse activity were determined by a PubMed search using the following search query: *gene name* AND glioma, *gene name* AND cancer, and *gene name* AND (brain OR “neurological disorder” OR neurodevelopment OR synapse).

### Data availability

GliomaScan and GICC data are available from the Database of Genotypes and Phenotypes (dbGaP) phs000652.v1.p1 and phs001319.v1.p1, respectively. The AGOG data are available from the authors upon reasonable request, with permission from all relevant human research ethics committees.

## Results

### Novel susceptibility regions

#### Stage 1: Hypothesis generation

Sixty-eight regions had a DEPTH PLO score of >2.0 for at least two of the three studies of the same sex and glioma type. Seven of these regions either contained a known susceptibility variant (*TERT*, *EGFR*, *CCDC26*, *CDKN2BAS*, *TP53*, and *RTEL1* regions) or were within 0.2 Mb of a known susceptibility variant (*POLR3B* region). The remaining 61 regions were candidate novel susceptibility regions, and all had a PLO of >95th percentile of the null distribution.

#### Stage 2: Region-based association testing

Association testing using GBJ found that 10 of the 61 novel regions from stage 1 had a GBJ *P* value of <0.05, for the same two studies, sex, and glioma type, as identified by the DEPTH analysis. One of the 61 regions (15q13.3) had a GBJ *P* value of <0.05 for the same two studies and the same glioma type but a different sex status to that identified by DEPTH (all-glioma male vs. all-glioma combined sex). This resulted in a total of 11 candidate novel susceptibility regions identified by GBJ analysis (Table 2). Two regions (2p25.1 and 7q31.33) remained significantly associated with glioma in at least one study after allowing for multiple testing adjustments (*P* < 0.001, 0.05/61). Regions 1p36.21, 15q13.3, 2q14.3, and 16p13.3 had consistent associations for one sex across at least three studies of the same glioma type.

**Table 2:** *P* values for candidate novel risk regions from the discovery, validation, and meta-analysis including the lowest *P* value SNP from each region

						Region-based analysis				SNP-based meta-analysis				Region-based meta-analysis	
						GBJ				Logistic regression				GBJ	MAGMA
						Discovery			Valid’n^a^						
						GliomaScan	AGOG	GICC	UCSF/Mayo				Meta-analysis	Meta-analysis^a^	Meta-analysis^a^
Locus	Start Mbp	End Mbp	Genes within the region	Glioma type	Sex at risk	*P* values				No. of SNPs in region	Lowest *P* value SNP in region	OR	*P* value	*P* values	
1p36.21	13.9	14.2	*PDPN*, *PRDM2*	All glioma	Male	**0.025**	**0.034**	**0.006**	0.470	1,017	rs35042965	0.81	1.54E−06***	0.002**	**0.020**
2p25.1	11.7	12.1	*GREB1*, *NTSR2*, *LPIN1*	GBM	Female	6.57E−05*	**0.001**	NA	0.830	1,372	rs72773920	2.15	**1.37E−04**	**0.035**	0.077
2q14.3	123.2	123.5	Intergenic	GBM	Male	**0.024**	**0.013**	NA	0.085^b^	979	rs958539	1.50	**0.002**	0.822	0.907
4q32.3	164.8	165.1	*MARCHF1*	Non-GBM	Both sexes	**0.008**	**0.035**	NA	0.812	1,068	rs114294504	1.79	**0.004**	0.731	0.254
5q35.2	174.4	174.9	*LINC01051*, *DRD1*, *SFXN1*	All glioma	Male	**0.003**	**0.007**	0.555	0.727^b^	1,806	rs13188939	0.79	**5.19E−04**	0.348	0.084
7q31.33	125.7	126.5	*GRM8*	GBM	Male	**0.036**	5.91E−05*	NA	1.000	2,330	rs11767389	1.29	**3.81E−04**	0.102	0.127
14q23.2	63.0	63.3	*KCNH5*	All glioma	Female	**0.019**	**0.005**	1.000	0.751	1,009	rs138459733	1.53	**0.005**	0.293	0.234
14q32.13	96.7	97.0	*BDKRB1*, *ATG2B*, *GSKIP*, *AK7*	All glioma	Both sexes	**0.021**	0.256	**0.003**	0.782	728	rs12050328	0.84	**1.62E-04**	**0.006**	0.004**
15q13.3	32.1	32.6	*OTUD7A*, *CHRNA7*	All glioma	Male	0.475	**0.006**	**0.014**	**0.039**	878	rs117034591	0.61	**0.001**	**0.006**	0.002**
16p13.3	7.0	7.4	*RBFOX1*	All glioma	Female	0.759	**0.018**	**0.004**	**0.009**	2,789	rs2346609	1.24	3.21E−07***	0.003**	0.001**
16p12.13	20.3	20.7	*GP2*, *UMOD*, *PDILT*, *ACSM5*, *ACSM2A*, *ACSM2B*, *ACSM1*, *THUMPD1*, *ACSM3*	All glioma	Both sexes	**0.022**	**0.002**	1.000	1.000^c^	1,172	rs4643331	1.65	**0.004**	0.911	0.562

Abbreviations: GBJ, generalized Berk-Jones statistic; Mbp, megabase pair as per hg19 genome build; meta-analysis, GliomaScan, AGOG, GICC, and UCSF/Mayo; Valid’n, validation dataset (UCSF/Mayo).

NA Glioma type information was unavailable for GICC and UCSF/Mayo sex-specific non-GBM summary statistics for 4q32.3 were unavailable.

Bold = *P* value < 0.05.

*, *P* value < multiple-testing adjusted threshold in discovery data (*P* = 0.001 = 0.05/61 regions).

**, *P* value < multiple-testing adjusted threshold in meta-analysis (*P* = 0.004 = 0.05/11 regions).

***, *P* value < multiple-testing adjusted threshold in SNP meta-analysis (*P* = 3.30E−06 = 0.05/15,148 SNPs).

a These analyses were conducted on summary statistics using 1000 genomes as a reference for linkage disequilibrium (LD) estimation.

b 2q14.3 and 5q35.2 regions reported *P* = 0.011 and *P* = 0.042 respectively using the combined sex validation data (UCSF/Mayo).

c 16p12.13 region reported *P* = 0.002 using female validation data (UCSF/Mayo).

#### Stage 3: Validation

Five of the 11 regions were associated with glioma in the validation dataset (UCSF/Mayo); however, only two of these five regions, 15q13.3 and 16p13.3, were found to be nominally associated (*P* = 0.039 and *P* = 0.009, respectively) with the same sex as that of the discovery data (Table 2). The three other regions (2q14.3, 5q35.2, and 16p12.13) were associated with glioma risk using the validation study but for a different sex to that of the discovery datasets (Supplementary Table S5). The regions 2q14.3 and 5q35.2 were associated with glioma risk for both sexes using the validation study (*P* = 0.011 and *P* = 0.042, respectively) while being associated with male risk in the discovery datasets. The 16p12.13 region was significantly associated with female glioma risk in the validation study (*P* = 0.002) after allowing for multiple testing adjustments but was associated with both sexes in the discovery datasets.

#### Stage 4: Meta-analysis

The effect size and association direction of the SNPs driving the region-based risk association for the 11 candidate susceptibility regions were obtained by conventional SNP-based logistic regression analysis of each of the three studies (GliomaScan, AGOG, and GICC). Across the individual studies for the 11 candidate regions, no SNP had a *P* value of genome-wide significance (*P* < 5.0 × 10^−8^) with the lowest *P* value being *P* = 3.2 × 10^−6^ for a SNP in the 16p13.3 region using AGOG data. For most regions, the SNPs driving the region-based risk association differed across the three studies.

An SNP-based meta-analysis of the summary statistics for the 11 candidate susceptibility regions of AGOG, GliomaScan, GICC, and UCSF/Mayo found two SNPs (rs2346609 and rs35042965) significantly associated with female and male glioma risks, respectively, after adjusting for multiple testing (Table 2). The variant rs2346609 is an intron variant of the *RBFOX1* gene and is significantly associated with female glioma susceptibility (OR = 1.24; *P* = 3.21 × 10^−7^). The variant is not associated with male risk and the two largest studies (Mayo/UCSF and GICC) had a sex difference in effect size for this variant (Table 3). The variant rs35042965 is an intergenic variant downstream of the *PRDM2* gene and is protective against male risk of glioma (OR = 0.81; *P* = 1.54 × 10^−6^). There is no association of this variant with female risk of glioma (Table 3).

**Table 3:** Odds ratio and *P* values for rs2346609 and rs35042965 by study and by sex

	Female				Male				
	OR	L95	U95	*P* value	OR	L95	U95	*P* value	*P* value sex difference
rs2346609 (chr16:7260432^a^) *RBFOX1*									
GliomaScan	1.20	1.00	1.43	0.0536	0.99	0.84	1.16	0.8613	0.1195
AGOG	1.17	0.94	1.46	0.1677	0.95	0.78	1.16	0.6419	0.1800
GICC	1.24	1.10	1.41	0.0008	0.99	0.88	1.10	0.7969	0.0066
Mayo/UCSF	1.31	1.11	1.56	0.0019	0.99	0.86	1.14	0.9173	0.0131
Meta-analysis	1.24			3.21E−07*	0.98			0.6474	
rs35042965 (chr1:14184325^a^) *PRDM2*									
GliomaScan	0.99	0.79	1.23	0.9103	0.91	0.74	1.10	0.3269	0.5709
AGOG	1.01	0.77	1.33	0.9322	0.75	0.58	0.97	0.0285	0.1180
GICC	0.98	0.83	1.14	0.7734	0.81	0.71	0.92	0.0016	0.0702
Mayo/UCSF	0.99	0.80	1.22	0.9203	0.77	0.65	0.92	0.0030	0.0744
Meta-analysis	0.99			0.7968	0.81			1.54E−06*	

*, *P* value < multiple-testing adjusted threshold in SNP meta-analysis (*P* < 3.3E−06 = 0.05/15,148 SNPs).

a Base pair location is hg19 build.

The GBJ set-based analysis of the meta-analysis results found two of the 11 regions (16p13.3 containing *RBFOX1* and 1p36.21 containing *PRDM2*) were significantly associated with female and male glioma risks, respectively, after adjusting for multiple testing (*P*_16p13.3_ = 0.003; *P*_1p36.21_ = 0.002; Table 2). Three other regions, 2p25.1, 14q32.13, and 15q13.3, were nominally associated with glioma risk using GBJ. Analysis of the same data using an alternative gene-based testing method (MAGMA) showed consistent results to that of the GBJ analysis with regions 16p13.3, 15q13.3, and 14q32.13 being significantly associated with glioma risk, whereas 1p36.21 was nominally associated with glioma risk (Table 2).

### Known susceptibility regions

The DEPTH PLO scores for the 34 known susceptibility regions for GliomaScan, AGOG, and GICC by sex and glioma type are shown in Supplementary Table S6A–S6C, and the GBJ *P*-values for *TERT*, *EGFR*, *CCDC26*, *CDKN2BAS*, *TP53*, and *RTEL1* gene regions are shown in Supplementary Table S7 for comparison.

## Discussion

The objective of this study was to identify novel genomic susceptibility regions of glioma using existing glioma case–control studies by analyzing regions of the genome for their association with glioma risk rather than individual SNPs. A region-based analysis may detect genomic regions where there are multiple independent SNPs associated with glioma risk but none of these SNPs individually meets genome-wide significance (5 × 10^−8^) using a conventional SNP-based analysis. To prioritize genomic regions, we first used a machine learning algorithm (DEPTH) to nominate candidate susceptibility regions for further formal association testing thereby reducing the multiple testing burden. DEPTH is a hypothesis-generating tool used in conjunction with conventional logistic regression or set-based analysis methods such as GBJ or MAGMA.

### Novel susceptibility regions

We identified 11 candidate susceptibility regions that suggested an association with all-glioma, GBM, or non-GBM (*P* < 0.05) across multiple studies (Table 2). No regions met our criteria for novel candidate susceptibility regions for astrocytoma or oligodendroglioma possibly because of the small sample sizes of these glioma types and the missing type data for GICC. The length of the 11 regions ranged from 300,000 to 800,000 bp per region and the number of SNPs analyzed within these regions ranged from 728 to 2,789 SNPs per region. Seven of the 11 candidate susceptibility regions contained genes previously reported as potential regulators of glioma tumor cell proliferation, and three other regions contained genes previously linked to tumor growth in other cancers (Table 4; Supplementary Table S8). Three of the 11 regions contain neurotransmitter receptor genes (7q31.33, 5q35.2, and 15q13.3).

**Table 4:** Association of the genes within the potential novel risk regions with the synapse, neurodevelopment, neurological disorders, glioma tumor cell growth, and other cancers according to a PubMed search

	Locus	Start (Mb)	End (Mb)	Genes	Protein	The synaptic region	Neurodevelopment	Neurological disorders	Glioma tumor cell growth	Other cancers
Combined Sex	4q32.3	164.8	165.1	*MARCHF1*	Membrane-associated ring-CH-finger 1					X
	14q32.13	96.7	97.0	*BDKRB1*	Bradykinin receptor B1				X	X
				*ATG2B*	Autophagy-related 2B				X	X
				*GSKIP*	GSK3B interacting protein					X
				*AK7*	Adenylate kinase 7					X
	16p12.13	20.3	20.7	*GP2*	Glycoprotein 2					X
				*UMOD*	Uromodulin					
				*PDILT*	Protein disulfide isomerase-like, testis expressed					
				*ACSM5*	Acyl-CoA synthetase medium-chain family member 5					
				*ACSM2A*	Acyl-CoA synthetase medium-chain family member 2A					
				*ACSM2B*	Acyl-CoA synthetase medium-chain family member 2B					
				*ACSM1*	Acyl-CoA synthetase medium-chain family member 1			X		X
				*THUMPD1*	THUMP domain containing 1			X		X
				*ACSM3*	Acyl-CoA synthetase medium-chain family member 3					X
Male	1p36.21	13.9	14.2	*PDPN*	Podoplanin				X	X
				*PRDM2*	Retinoblastoma protein-interacting zinc finger gene 1				X	X
	2q14.3	123.2	123.5	Intergenic						
	5q35.2	174.4	174.9	*LINC01951*	Noncoding RNA					
				*DRD1*	Dopamine receptor D1	X	X	X	X	X
				*SFXN1*	Sideroflexin 1				X	
	7q31.33	125.7	126.5	*GRM8*	Metabotropic glutamate receptor 8	X			X	X
	15q13.3	32.1	32.6	*OTUD7A*	OUT deubiquitinase 7A			X		X
				*CHRNA7*	Cholinergic receptor nicotinic alpha 7 subunit	X		X		
Female	2p25.1	11.7	12.1	*GREB1*	Growth regulation by estrogen in breast cancer 1					X
				*NTSR2*	Neurotensin receptor type 2				X	X
				*LPIN1*	Lipin 1					X
	14q23.2	63.0	63.3	*KCNH5*	Potassium voltage-gated channel subfamily H member 5	X			X	X
	16p13.3	7.0	7.4	*RBFOX1*	RNA binding fox-1 homolog 1		X	X	X	

Abbreviation: Mb, megabase as per h19 genome build.

Of the 11 candidate susceptibility regions, two regions were consistently associated with glioma risk across multiple studies and methods, 16p13.3 an intronic region of the *RBFOX1* gene, and 1p36.21 containing the genes *PDPN* and *PRDM2*. Both regions were nominally associated with glioma risk across three of the four studies and were significantly associated with glioma risk for the meta-analysis after adjusting for multiple testing (Table 2). Both regions contained SNPs that were significantly associated with glioma risk after adjustment for multiple testing. Our discussion will focus on these two potential susceptibility regions; however, consideration will also be given to the regions 14q32.13 and 15q13.3, which were nominally associated with glioma risk across multiple individual studies and the meta-analysis. Considering recent seminal studies regarding the effect of synaptic communication on glioma tumor progression (40–43), we will also discuss the three regions that contain neurotransmitter receptor genes (7q31.33, 5q35.2, and 15q13.3).

#### Region 16p13.3 (intronic region of *RBFOX1*)

The 16p13.3 region contains the RNA binding fox-1 homolog 1 (*RBFOX1*) gene and is associated with all-glioma female risk. *RBFOX1* regulates tissue-specific alternative splicing, has been associated with neurodevelopmental disorders and seizures, regulates the brain blood–tumor barrier (44), and is a germline risk locus for genetic generalized epilepsy (45). Loss of *RBFOX1* function promotes gliomagenesis, and low *RBFOX1* expression in glioma tissues is associated with poor survival (44). A recent Taiwanese GWAS of 195 glioma cases identified the *RBFOX1* variant rs8044700 as a potential glioma risk variant for the Han Chinese (OR = 2.36; *P* = 2.4 × 10^−5^; ref. 46). The variant rs8044700 is upstream of the 400,000-bp risk region identified by our study and was not associated with glioma in our analyses. The region identified in our analysis is an intronic region of *RBFOX1* located between 7.0 and 7.4 Mbp (hg19 build). The region is associated with female all-glioma risk and the lowest *P* value SNP identified by the meta-analysis, rs2346609, showed consistent effect size across all four studies and had a significant sex difference in effect size in two of the four studies (Table 3). The variant shows no association with male risk.

#### Region 1p36.21 (containing *PDPN* and *PRDM2*)

The 1p36.21 region contains retinoblastoma protein-interacting zinc-finger (*PRDM2*) and podoplanin (*PDPN*) genes. *PRDM2* encodes a histone methyltransferase enriched in the prefrontal cortex (47). It is a known tumor suppressor of gliomagenesis (48) and its expression is mediated by estrogen (47). The *PDPN* gene encodes a cell surface protein that mediates platelet adhesion, aggregation, and secretion during embryonic development of the vasculature and has been linked to tumor invasion and progression in many cancers, including glioma, where it may contribute to the immunosuppressive microenvironment of GBM (49). This region was associated with all-glioma male risk using GliomaScan, AGOG, and GICC (Figs. 2 and 3), with evidence of association with male GBM risk using GliomaScan (Supplementary Table S9). The UCSF/Mayo summary data did not show evidence of an association with male risk, but there was weak evidence of an association with female risk (Supplementary Table S5). The lowest *P* value SNP identified by the meta-analysis, rs35042965, showed a protective association for male glioma risk with consistent effect size across all four studies (Table 3). The same SNP reported no association with female risk. There was no statistically significant sex difference in effect size for this SNP but three of the four studies did report a sex difference *P* value of ∼0.10. The SNP is located ∼30,000 bp downstream of *PRDM2*.

#### Region 15q13.3 (containing *OTUD7A* and *CHRNA7*)

The 15q13.3 region contains the deubiquitinase 7A (*OTUD7A*) and alpha7 neuronal nicotinic acetylcholine receptor (*CHRNA7*) genes. The *CHRNA7* gene belongs to a family of ion channels that mediates fast signal transmission at the synapse, is associated with multiple neurological and psychiatric disorders, and plays a role in tumor progression in multiple cancers (50, 51). Deletion of the 15q13.3 region containing *OTUD7A* and *CHRNA7* is associated with schizophrenia with loss of function of *OTUD7A* resulting in impaired synapse function and development (51). This region was associated with male all-glioma risk. No SNP in the region met multiple testing *P* value thresholds in the meta-analysis.

#### Region 14q32.13 (containing *BDKRB1*, *ATG2B*, *GSKIP*, and *AK7*)

The 14q32.13 region contains bradykinin receptor B1 (*BDKRB1*), autophagy-related 2B (*ATG2B*), GSK3B interacting protein (*GSKIP*), and adenylate kinase 7 (*AK7*) genes. *ATG2B* is an autophagy gene that was previously identified as a potential GBM risk gene in a hospital-based case–control study of 174 cases (52). This region was associated with all-glioma combined-sex risk. There was no association with glioma risk using the UCSF/Mayo data. No SNP in the region met multiple testing *P* value thresholds in the meta-analysis.

#### Regions contained neurotransmitter receptor genes

Recent seminal studies have identified neuronal glioma cell communication as a driver of tumor growth through synaptic signaling (40–43). Three regions in Table 2 contain neurotransmitter receptor genes, the 5q35.2 region contains the dopamine receptor D1 (*DRD1*), the 15q13.3 region contains the alpha7 neuronal nicotinic acetylcholine receptor (*CHRNA7*) gene and the 7q31.33 region contains the metabotropic glutamate receptor 8 (*GRM8*) gene. The 15q13.3 region is described above. The 7q31.33 region was identified as a male GBM susceptibility region. GBM-type data were unavailable for GICC, and there was no evidence of glioma risk association of the 7q31.33 region using the UCSF/Mayo data. The *GRM8* gene is a breast cancer oncogene, and its expression is known to inhibit glioma tumor cell proliferation (53). Venkataramani and colleagues (40) found neural activity via glutamatergic synaptic input drives glioma invasion and progression, and they were able to block glutamatergic synaptic communication between neurons and glioma cells with an approved antiepileptic drug attenuating glioma progression in mice. The 5q35.2 region contains the dopamine receptor D1 (*DRD1*) and long noncoding RNA (*LINC01951*) genes. Dopamine receptors are associated with several neurological disorders and play a regulatory role in motor activity and neurogenesis (54). *DRD1* is highly expressed in glioma tumors and mediates tumor growth (55). This region was nominally associated with all-glioma risk region using all four studies; however, it was associated with different sexes across studies. The region was associated with male risk using AGOG and GliomaScan, female risk using GICC, and risk for both sexes using UCSF/Mayo data, suggesting that this may be a susceptibility region for both sexes. Our findings of three potential susceptibility regions containing neurotransmitter receptors warrant further studies on the role of synaptic genes in genetic susceptibility to glioma.

#### Aggregated data analysis

We merged the individual-level data for GliomaScan, AGOG, and GICC into an aggregated dataset to assess whether the GBJ *P* value for the 11 candidate regions had stronger or weaker evidence of association with glioma after aggregation. The aggregated data were not used for discovery purposes, as the three studies differed in their data collection protocols and genotyping. The GBJ analysis of the aggregated data after adjusting for sex, age, 7PCs, and study found that the GBJ *P* value attenuated relative to the individual study GBJ *P* values (Supplementary Table S5). Investigation of this attenuation using SNP-based logistic regression analysis of the 11 regions revealed several possible causes. First, the SNPs driving the association signal of the region differed across individual studies. This could arise if there are multiple markers correlated with one or multiple unknown causal variants owing to differences in LD among Europeans. Second, some SNPs associated with glioma risk in one study were missing from the other studies and were eliminated during the data-merging QC process. Third, in some instances, a low *P* value SNP across all studies had an opposing risk direction in one study (e.g., protective in one study and harmful in the other). Although care was taken to ensure consistency in risk allele and strand alignment across the studies upon data merging, it is possible that strand-ambiguous SNPs (A/T and C/G alleles) may have different strand alignments across the studies. Finally, we cannot exclude the possibility of false positive signals for the same region in all datasets.

### Known susceptibility regions

To assess DEPTH’s capability to generate suitable candidate susceptibility regions for subsequent association testing, we applied DEPTH to the 34 known susceptibility regions, and 91%, 82%, and 74% of the regions had a PLO of >1.0 (10 times more likely to be associated with glioma) using GliomaScan, GICC, and AGOG, respectively (Supplementary Table S6A–S6C). A higher detection rate for GICC may have been possible if we had access to GICC glioma-type data, as many of the 34 known regions are type-specific (8). For the well-replicated susceptibility regions containing *TERT*, *EGFR*, *CCDC26*, *CDKN2BAS*, *TP53*, and *RTEL1*, DEPTH reported a PLO of >2.0, for at least two of the three studies. PLO scores for *TERT* and *CCDC26* were as high as 18.4 and 20.0, respectively, with *CCDC26* PLO scores showing greater evidence of female risk, supporting the findings of previous sex-specific SNP-based GWASs (Supplementary Table S7; refs. 11, 29).

### Limitations

Limitations of our study are as follows: (i) The absence of glioma type for the GICC data and the small astrocytoma and oligodendroglioma sample sizes of AGOG and GliomaScan limited the discovery of glioma type-specific susceptibility regions; (ii) we only examined regions with DEPTH PLO of ≥2.0, although there may be regions with significant GBJ *P* values in the PLO range of 1.0 to 2.0, and these will be the focus of future investigations; (iii) false negatives may be among the 50 of the 61 DEPTH-identified candidate novel regions that failed to meet GBJ *P* value significance thresholds as DEPTH’s decision tree analysis accounts for SNP interactions and detects nonlinear associations that may be missed by the GBJ linear model; (iv) the UCSF/Mayo summary statistic data contains controls overlapping with those of GICC, so the two studies are not entirely independent; (v) for the GBM and non-GBM potential susceptibility regions, validation in the UCSF/Mayo data was performed using IDHwt and IDH mutant data as a proxy for GBM and non-GBM, respectively; therefore, the UCSF/Mayo GBJ results for GBM/non-GBM data may not be entirely comparable to that of AGOG, GliomaScan, or GICC; and lastly, (vi) the application of our findings may be restricted to individuals of European ancestry.

In conclusion, our study identified 11 genomic regions of which two regions, 1p36.21 and 16p13.3, warrant further investigation as genetic susceptibility regions for male and female glioma risk, respectively. We demonstrated that innovative statistical methods that analyze groups of SNPs rather than individual SNPs may detect novel genetic susceptibility regions using existing glioma datasets. This is an important finding considering the difficulty in increasing sample sizes for this rare and debilitating cancer. Our findings suggest that genetic susceptibility to glioma may differ by sex, highlighting the importance of sex-specific analyses in glioma research. We also propose further association studies for regions containing synapse-related genes to assess their possible association with glioma risk.

## Supplementary Material

Supplementary Figure S1 Supplementary Figure S1 is a stylised illustration of DEPTH’s use of sliding windows to identify genomic susceptibility regions.

Supplementary Figure S2 Supplementary Figure S2 shows the estimation of genetic ancestry of AGOG, GliomaScan and GICC samples by way of scatter plot of PC1 versus PC2.

Supplementary Table S1 Supplementary Table S1 shows the age characteristics of the UCSF/Mayo data (validation data).

Supplementary Table S2 Supplementary Table S2 shows the number of samples excluded from the GliomaScan data and reasons for exclusion.

Supplementary Table S3 Supplementary Table S3 shows the number of samples excluded from the AGOG autosomal data (S3a) and X-chromosome data (S3b) and reasons for exclusion.

Supplementary Table S4 Supplementary Table S4 shows the number of samples excluded from the GICC data and reasons for exclusion.

Supplementary Table S5 Supplementary Table S5 shows the generalised Berk-Jones statistic (GBJ) *p*-values for the candidate novel susceptibility regions for glioma by study and sex.

Supplementary Table S6 Supplementary Table S6 shows the DEPTH posterior log odds in favour of association (PLO) score and the logistic regression p-value of the lowest p-value variant for each of the 34 known glioma risk regions using GliomaScan (S6a), AGOG (S6b), and GICC (S6c).

Supplementary Table S7 Supplementary Table S7 shows the DEPTH posterior log odds in favour of association (PLO) score and generalised Berk-Jones statistic (GBJ) *p*-value for TERT, EGFR, CCDC26, CDKN2BAS, TP53 and RTEL regions.

Supplementary Table S8 Supplementary Table S8 summarizes the supporting evidence for the potential novel glioma susceptibility regions.

Supplementary Table S9 Supplementary Table S9 shows the generalised Berk-Jones statistic (GBJ) *p*-values for the potential novel glioma susceptibility regions by study, glioma type and sex.
