# Supplementary material for: Region-Based Analyses of Existing Genome-Wide Association Studies Identifies Novel Potential Genetic Susceptibility Regions for Glioma
Source: Cancer Res Commun. 2024 Nov 12;4(11):2933–46. doi: 10.1158/2767-9764.CRC-24-0385 (PMC11555644; doi:10.1158/2767-9764.CRC-24-0385)
Supplement: Supplementary Figure S1 — is a stylised illustration of DEPTH’s use of sliding windows to identify genomic susceptibility regions. [file crc-24-0385_supplementary_figure_s1_suppsf1.pdf]

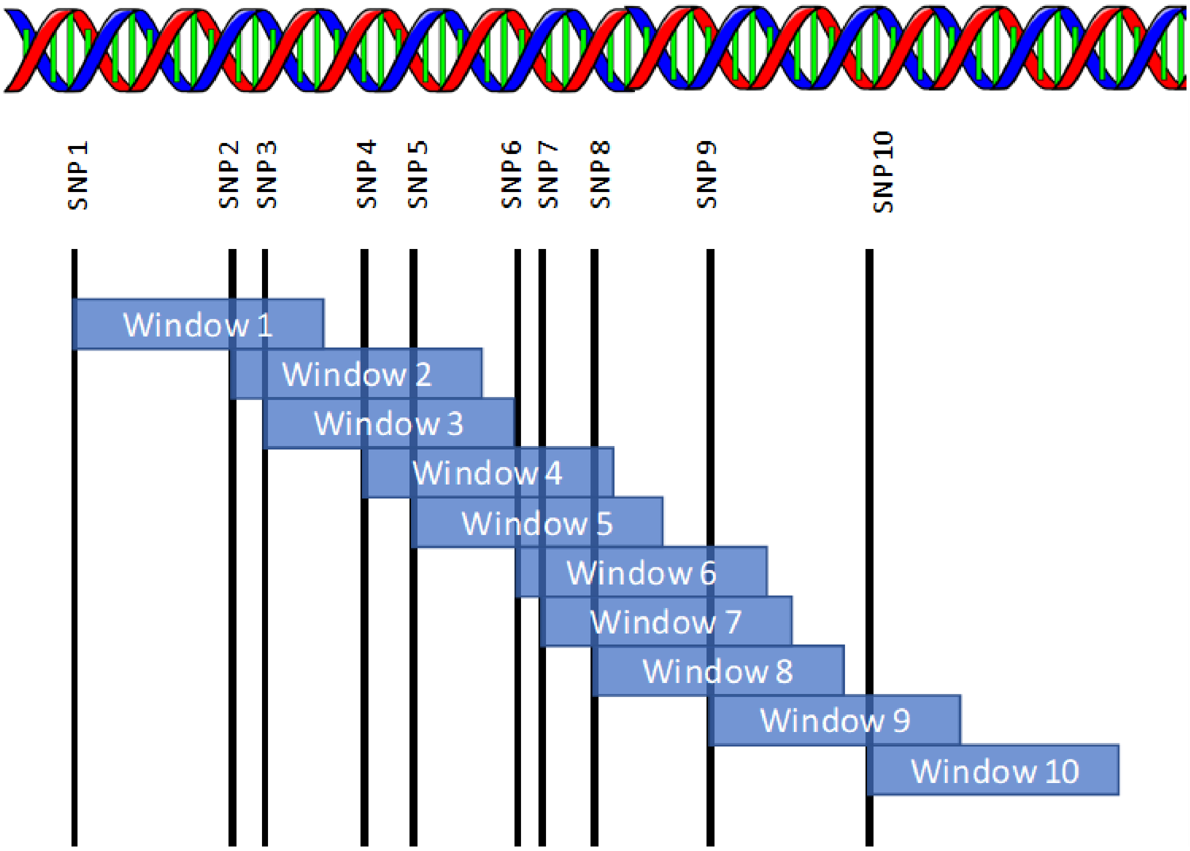

Supplementary Figure S1. Stylised illustration of DEPTH's use of windows of SNPs to identify genomic susceptibility regions. In Window 1, DEPTH compares the allele frequencies between cases and controls for SNP 1, 2 and 3, creates a decision tree and reports the association score (Bayesian posterior log odds in favour of association) for that window. The algorithm then slides onto Window 2 and creates a decision tree based on the case versus control allele frequencies of SNPs 2, 3, 4 and 5 and reports the association score for Window 2 and then so on for Window 3, 4, 5 etc. The posterior log odds for each window can then be plotted on a UCSC graph and regions with high association scores are easily identifiable as peaks.
