# Supplementary material for: Region-Based Analyses of Existing Genome-Wide Association Studies Identifies Novel Potential Genetic Susceptibility Regions for Glioma
Source: Cancer Res Commun. 2024 Nov 12;4(11):2933–46. doi: 10.1158/2767-9764.CRC-24-0385 (PMC11555644; doi:10.1158/2767-9764.CRC-24-0385)
Supplement: Supplementary Figure S2 — shows the estimation of genetic ancestry of AGOG, GliomaScan and GICC samples by way of scatter plot of PC1 versus PC2. [file crc-24-0385_supplementary_figure_s2_suppsf2.pdf]

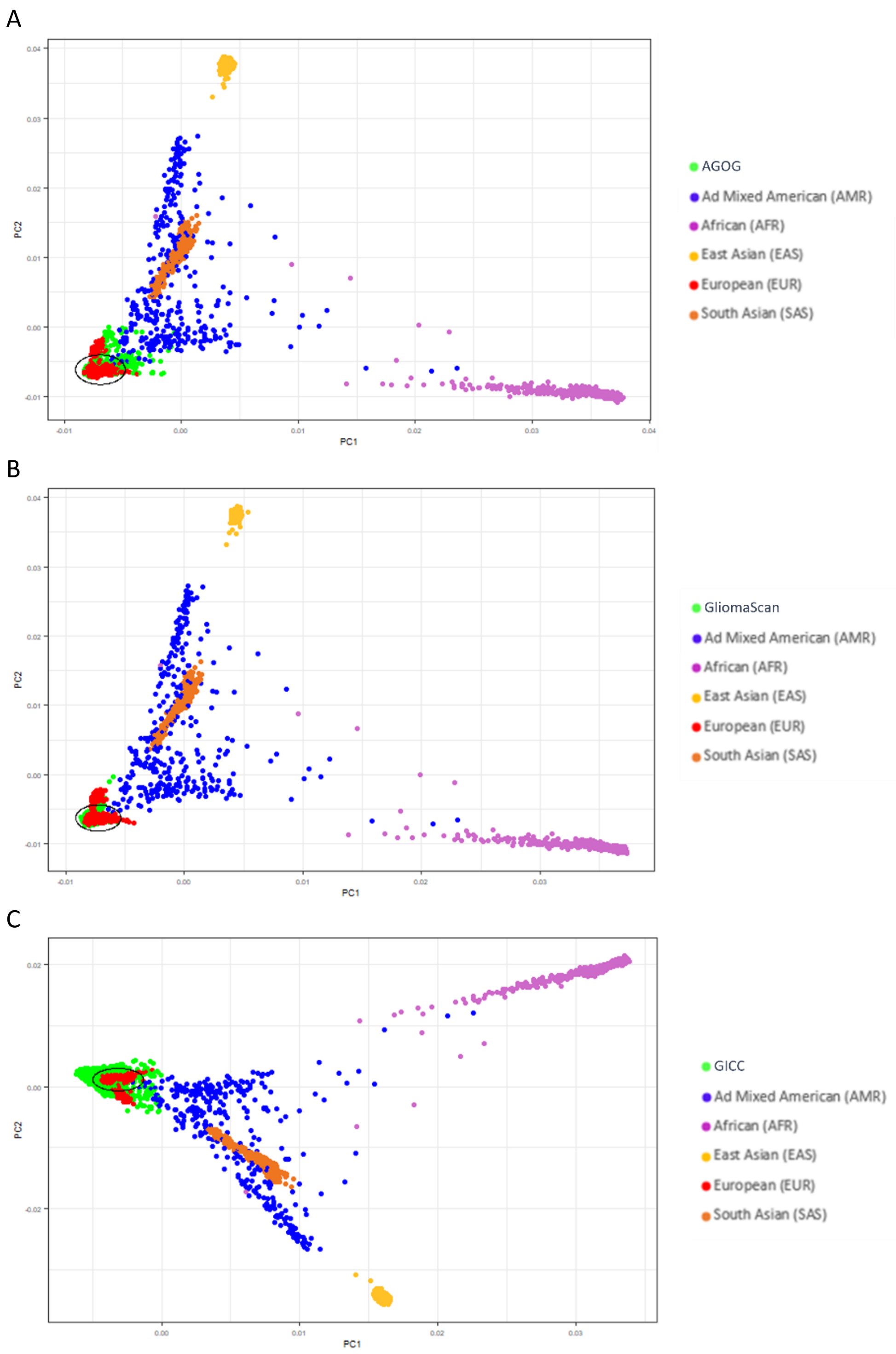

Supplementary Figure S2. Estimation of ancestry of the A) AGOG, B) GliomaScan and C) GICC study samples with reference to the 1000 genome reference by way of scatter plot of PC1 versus PC2. The black circle represents the centre of the known European reference samples. AGOG, GliomaScan and GICC samples are represented by green markers many of which are visually obscured by the overlayed red European reference sample. PC = principal component. Plots were produced using R software by Hanna Meyer https://cran.r-project.org/web/packages/plinkQC/vignettes/AncestryCheck.pdf
